# Bundle geodesic convolutional neural network for diffusion-weighted imaging segmentation

**DOI:** 10.1117/1.JMI.9.6.064002

**Published:** 2022-11-17

**Authors:** Renfei Liu, François Lauze, Kenny Erleben, Rune W. Berg, Sune Darkner

**Affiliations:** a University of Copenhagen, Department of Computer Science, Copenhagen, Denmark; b University of Copenhagen, Department of Neuroscience, Copenhagen, Denmark

**Keywords:** G-convolutional neural networks, group convolution, geometric deep learning, diffusion-weighted imaging

## Abstract

**Purpose:**

Applying machine learning techniques to magnetic resonance diffusion-weighted imaging (DWI) data is challenging due to the size of individual data samples and the lack of labeled data. It is possible, though, to learn general patterns from a very limited amount of training data if we take advantage of the geometry of the DWI data. Therefore, we present a tissue classifier based on a Riemannian deep learning framework for single-shell DWI data.

**Approach:**

The framework consists of three layers: a lifting layer that locally represents and convolves data on tangent spaces to produce a family of functions defined on the rotation groups of the tangent spaces, i.e., a (not necessarily continuous) function on a bundle of rotational functions on the manifold; a group convolution layer that convolves this function with rotation kernels to produce a family of local functions over each of the rotation groups; a projection layer using maximization to collapse this local data to form manifold based functions.

**Results:**

Experiments show that our method achieves the performance of the same level as state-of-the-art while using way fewer parameters in the model (<10%). Meanwhile, we conducted a model sensitivity analysis for our method. We ran experiments using a proportion (69.2%, 53.3%, and 29.4%) of the original training set and analyzed how much data the model needs for the task. Results show that this does reduce the overall classification accuracy mildly, but it also boosts the accuracy for minority classes.

**Conclusions:**

This work extended convolutional neural networks to Riemannian manifolds, and it shows the potential in understanding structural patterns in the brain, as well as in aiding manual data annotation.

## 1 Introduction

Studies for magnetic resonance diffusion-weighted imaging (DWI) data have small sample sizes in general due to the lack of manually annotated data. For machine learning techniques, this poses a major challenge. However, learning general patterns from a limited amount of data while producing promising results is possible by conducting a special Geodesic convolutional neural network (G-CNN) architecture that takes advantage of the geometry of the data. In general, to define convolution, the underlying space must have a group structure or be a homogeneous space of a group. This is not the case for most curved spaces. But even when it is, like the spheres on which local DWI signals are defined, this often imposes a certain type of filter. Instead, we present a framework that focuses on building a neural network (NN) for data on Riemannian manifolds with some simple form of orientation invariance, and we take DWI as the main application. There are a series of proposals trying to generalize a $R^{2}$ convolutional neural network to curved spaces, yet in our case, rotational invariance is a desirable property we want in the design and our goal is to be able to understand spherical patterns up to rotations. We propose a general architecture for extracting and filtering local orientation information of data defined on a manifold that allows us to learn similar orientation structures which can appear at different locations on the manifold. Reasonable manifolds have local orientation structures—rotations on tangent spaces. Our architecture lifts data to these structures and performs local filtering on them, after which it collapses them back to obtain filtered features on the manifold. This provides both rotational invariance and flexibility in design, without having to resort to complex embeddings in Euclidean spaces. Our contribution in this work is as follows:

• Instead of using Fourier-type methods such as irreducible representations as is done in literature, we directly perform convolution numerically on the surface as is done in classical CNNs in image analysis, which is far more light-weight;
• We lift the spherical function locally with SO(2) actions instead of lifting it to the full SO(3) group as is usually done in literature, which makes our method a more general case that is applicable on manifolds that are not spheres;
• We provide an explicit construction of the architecture for DWI data and show very promising results for this case including learning and generalizing patterns of a dataset from only one scan.

This work is an extension of our previous publication.^1^

## 2 Related Work

The importance of the extraction of rotationally invariant features beyond fractional anisotropy^2^ has been recognized in series of DWI works. Caruyer and Verma^3^ developed invariant polynomials of spherical harmonic (SH) expansion coefficients, and discussed their application in population studies. Schwab et al.^4^ proposed a related construction using eigenvalue decomposition of SH operators. Novikov et al.^5^ and Zucchelli et al.^6^ argued their usefulness for understanding microstructures in relation to DWI.

There is though a vast growth in literature on deep learning (DL) for non-flat data or more complex group actions than just translations. Masci et al.^7^ proposed an NN on surfaces that extracts local rotationally invariant features. A nonrotationally invariant modification was proposed in Boscaini et al.^8^ On the other hand, convolution generalizes to more group actions than just translation, and this has led to group-convolution neural networks for structures where these operations are supported, especially Lie groups themselves and their homogeneous spaces.^9^^–^^15^ Global equivariance is often sought but proved complicated or even elusive in many cases when the underlying geometry is nontrivial.^16^ An elementary construction on a general manifold is proposed by Schonsheck et al.^17^ via a fixed choice of geodesic paths used to transport filters between points on the manifold, ignoring the effects of path dependency (holonomy when paths are geodesics). The removal of this dependency can be obtained by summarizing local responses over local orientations, which is what was done by Masci et al.^7^ On the other hand, Cohen et al.^18^ lifted spherical functions to the 3D-rotation group SO(3) and used a generalization of Fourier transform on it to perform convolution. To explicitly deal with holonomy, Sommer and Bronstein^19^ proposed a convolution construction on manifolds based on stochastic processes via the frame bundle, but it is, at this point, still very theoretical.

A number of works have applied DL to DWI as well, due to the unique structure of the data, as orientation responses. Golkov et al.^20^ built multilayer perceptrons in q-space for kurtosis and NODDI mappings. Wasserthal et al.^21^ proposed a U-net inspired structure for tract segmentation, while Sedlar et al.^22^ proposed a spherical U-net for neurite orientation. To take into account the spherical structure of the DWI data and the homogeneous structure of the sphere, Chakraborty et al.^23^ proposed an rotation equivariant construction inspired by Cohen et al.^18^ for disease classification. Müller et al.^24^ propose a sixth-D, 3D space, and q-space NNs with roto-translation/rotation equivalence properties.

In this work, we are interested in rotationally invariant features, thus we take a path closer to Schonsheck et al.^17^ and Masci et al.,^7^ we actually lift functions to functions on the *bundle* of tangent space rotations of our manifolds, a two-dimensional manifold, as opposed to Cohen et al.^18^ where the lifting results in functions on SO(3)—a three-dimensional manifold. Then, we add one or more extra local group convolution layers before summarising the data and eliminating path dependency. The proposed construction thus applies to oriented Riemannian manifolds, and no other structure (e.g., homogeneous or symmetric space) is used.

## 3 Method

All along this section our reference to Riemannian Geometry can be found in the Do Carmo’s classical book *Riemannian Geometry*.^25^ CNNs are generally described and implemented in terms of correlation rather than convolution, and we follow this convention as well in this section. Bekkers et al.^14^ used the fact that SE(2) acts on $R^{2}$ to lift 2D (vector-values) images to $R^{2}\times S^{1}$ via correlation kernels. This is not, in general, the case when $R^{2}$ is replaced by a Riemannian manifold, where there is no obvious way to define these operations. One can, however, overcome this situation via a somewhat more complex construction. Therefore, we assume in the sequel that we are given a complete orientable Riemannian manifold of dimension n, this will be the sphere $S^{2}$ in our case. We assume that the injectivity radius i(M) of M is strictly positive. As usual, the tangent space at a point x∈M is $T_{x}M$. An image is a function $f=(f_{1}\ldots ,f_{N_{c}})\in L^{2}(M,R^{N_{c}})$, where $N_{c}$ is the number of channels.

Operations will be performed by lifting the function to tangent spaces and kernels are defined on tangent spaces. The exponential map ${\mathrm{Exp}}_{x}:T_{x}M\to M$ allows us to lift f to $T_{x}M$ by setting $f_{x}={\left(f_{i,x}\right)}_{i}\equiv f\circ {\mathrm{Exp}}_{x}$.

### 3.1 Layer Definitions

#### 3.1.1 Lifting layer

We first define transportable filters on tangent spaces to replace CNN’s kernels. These filters will also be called kernels. To start with, a “pointed kernel” will be a function $k=(k_{1},\ldots ,k_{N_{c}})\in L^{2}(T_{x_{0}}M,R^{N_{c}})$, at a “base point $x_{0}$.” We assume that $\mathrm{Supp}(k)\in B_{x_{0}}(0,r)$, 0<r≤i(M), the ball of center 0 and radius r in $T_{x_{0}}M$. A piece-wise smooth path γ:[0,1]→M, joining $x_{0}$ to x defines, via the Levi–Civita connection of M, *a parallel transport* $P_{\gamma }:T_{x_{0}}M\to T_{x}M$, and this is an isometry. We set $k_{\gamma }\equiv k\circ P_{\gamma }^{-1}$. In general, another smooth path δ:[0,1]→M joining $x_{0}$ and x defines another parallel transport $P_{\delta }:T_{x_{0}}M\to T_{x}M$ and $P_{\gamma }\circ P_{\delta }^{-1}$ is a rotation R of $T_{x}M$, i.e., an element of $\mathrm{SO}(T_{x}M)$. It follows that $k_{\delta }=k_{\gamma }\circ R$. The γ-lift of f by k is the function

$$(k{⋆}_{\gamma }f)(S)=\overset{N_{c}}{\underset{i=1}{\sum }}\int_{T_{x}M}\kappa_{i,\gamma }(S^{-1}v)f_{i,x}(v)dv,\;S\in \mathrm{SO}(T_{x}M).$$ (1)

Note that because $\mathrm{Supp}(k)\in B_{x_{0}}(0,r)$, this integral is defined on $B_{x}(0,r)$ and ${\mathrm{Exp}}_{x}$ is a diffeormorphism from this domain to the geodesic ball B(x,r)⊂M.

**Fig. 1:**
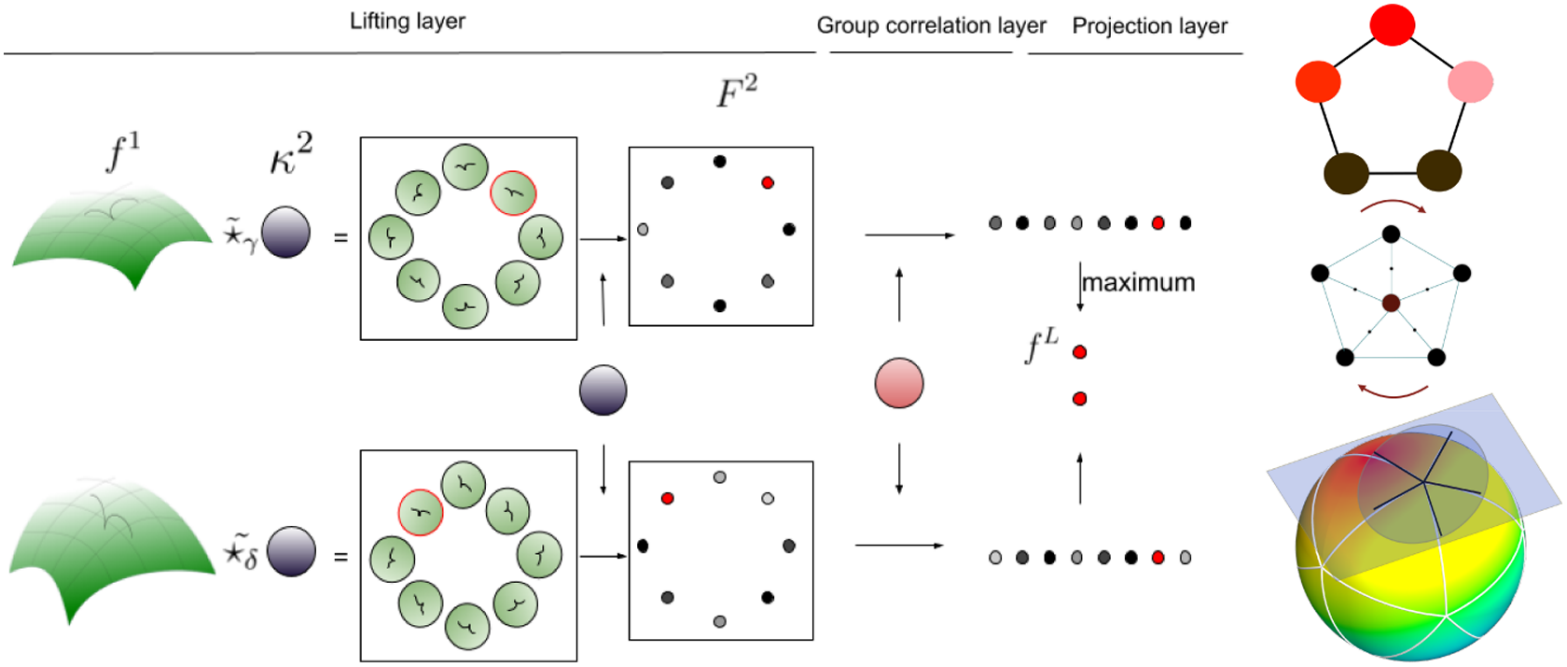
In the figure on the left, the top row shows the lifting kernel $\kappa^{\left(2\right)}$ applied at a point on the manifold, resulting in an image $F^{\left(2\right)}$ defined on SO(2) as in Eq. (2). The function is first mapped onto the tangent space of the point of interest via the exponential map, and $\kappa^{\left(2\right)}$ is convolved with the mapped function to get $F^{2}$. Group correlation is then performed on the resulting image, followed by the projection layer, from which we get rotationally invariant responses. The bottom row shows the same process but with a different kernel parallel transport, illustrating that the responses of the convolutional layers are simply rotated. In the figure on the right, the bottom row shows $S^{2}$ with a regular icosahedric tessellation and a tangent plane at one of the vertices and five sampled directions. The disk represents the kernel support. The middle row shows the actual discrete kernel used, with the 2π/5 rotations and the top row is represents the lifted function on the discrete rotation group.

Now we choose, for each x in M, a smooth path $\gamma_{x}$ that joins $x_{0}$ and x. As M is complete, we can, for instance, choose a family $\Gamma ={\left(\gamma_{x}\right)}_{x}$ of minimizing geodesics. The mapping

$$f\mapsto F=(k{⋆}_{\gamma_{x}}f{)}_{\gamma_{x}\in \Gamma },\;F(x):\;R\in SO(T_{x}M)\mapsto (k{⋆}_{\gamma_{x}}f)(R)\in R,$$ (2)

lifts a M-image to the bundle of rotations of M (we refer to Gallier et al.^26^ chap. 9 for a definition of bundles in differential geometry), denoted by SO(TM) in the sequel ($SO{\left(TM\right)}_{x}=SO(T_{x}M)$) as in Fig. 1. This lifting depends on the choice of the base point $x_{0}$ and the choice of paths from $x_{0}$ to any point x of M. The lifting layer at level (ℓ−1) takes a function $f:M\to R^{N_{\ell -1}}$ and uses $N_{\ell }$ kernels $\left(k^{1(\ell )},\ldots ,k^{N_{\ell }(\ell )}\right)$ to produce

$$F^{\left(\ell \right)}={\left(k^{1(\ell )}{⋆}_{\gamma_{x}}f^{\left(\ell -1\right)},\ldots ,k^{N_{\ell }(\ell )}{⋆}_{\gamma_{x}}f^{\left(\ell -1\right)}\right)}_{\gamma_{x}}.$$ (3)

#### 3.1.2 Group correlation layer

The object F defined in Eq. (2) is a function on the total space of the bundle (SO(TM)) (Gallier et al.^26^ chap. 9), supposed square-integrable ($F\in L^{2}(SO(TM))$).

The situation is more complex than the one described in Bekkers et al.,^14^ as there is actually no reason that one can find a “continuous family” of paths $x_{0}↝x$, ∀  x∈M. An important example to us: if M is the sphere $S^{2}$, one can take $\gamma_{x}$ to be a minimizing geodesic between $x_{0}$ and s. It is unique, except when $x=-x_{0}$, where there are infinitely many of them.

Let K be an element of $L^{2}(T_{x_{0}}M)$. The parallel transport of K along the path γ is $K_{\gamma }(R)=K(P_{\gamma }^{-1}{\mathrm{RP}}_{\gamma })$, as $P_{\gamma }^{-1}{\mathrm{RP}}_{\gamma }\in \mathrm{SO}(T_{x_{0}}M)$. The correlation $F(x)⋆K_{\gamma_{x}}$ is the group-theoretic one

$$F(x)⋆K_{\gamma_{x}}(S)=\int_{\mathrm{SO}(T_{x}M)}F(x)(R)K_{\gamma_{x}}(S^{-1}R)dR,$$ (4)

with *dR* the bi-invariant Haar measure on $\mathrm{SO}(T_{x}M)$. In general, we consider objects that are a bit more complicated. Instead of F being a section of $L^{2}(SO(TM))$, it is taken as a section of $L^{2}(SO(TM){)}^{N_{l}}$, meaning we have $N_{l}$ channels, $F(x)=(F(x{)}_{1},\ldots ,F(x{)}_{N_{l}})\in L^{2}(\mathrm{SO}(T_{x}M),R^{N_{l}})$, and K also has $N_{l}$ channels, $K=(K_{1},\ldots ,K_{N_{l}})\in L^{2}(\mathrm{SO}(T_{x_{0}}),R^{N_{l}})$ and we replace Eq. (4) with

$$F(x)⋆K_{\gamma_{x}}(S)=\overset{N_{l}}{\underset{i=1}{\sum }}\int_{\mathrm{SO}(T_{x}M)}F(x{)}_{i}(R)K_{i,\gamma_{x}}(S^{-1}R)\mathrm{dR}.$$ (5)

The group correlation layer at level ℓ takes a section F of $L^{2}(SO(TM){)}^{N_{l}}$, and uses $N_{\ell +1}$ kernels $\left(K_{1}^{\left(\ell +1\right)},\ldots K_{N_{\ell +1}}^{\left(\ell +1\right)}\right)$ to produce

$$F^{\left(\ell +1\right)}={\left(F^{\left(\ell \right)}(x)⋆K_{1,\gamma_{x}}^{(\ell +1),},\ldots ,F^{\left(\ell \right)}(x)⋆K_{N_{\ell +1},\gamma_{x}}^{\left(\ell +1\right)}\right)}_{x\in M}.$$

#### 3.1.3 Projection layer

The base point and path dependency in the lifting and group correlation layer definitions appear problematic. We can, however, reproject the results from these layers to standard functions on M, eliminating this dependency. The only condition is that the same family of paths is used both in the lifting and group correlation layers to parallel transport the kernels.

Indeed, from what precedes, two γ- and δ-lifts, though in general distinct, obey the simple relation

$$(k{⋆}_{\gamma }f)(S)=(k{⋆}_{\delta }f)(\mathrm{SR}),\;R=P_{\gamma }\circ P_{\delta }^{-1}.$$ (6)

A direct computation shows that $K_{\delta }(S)=K_{\gamma }(R^{-1}\mathrm{SR})$

$$\left((k{⋆}_{\delta }f)⋆K_{\delta })(S)=\int_{\mathrm{SO}(T_{x}M)}(k{⋆}_{\delta }f)(T)K_{\delta }(S^{-1}T)\mathrm{dT}\;=\int_{\mathrm{SO}(T_{x}M)}(k{⋆}_{\gamma }f)(\mathrm{TR})K_{\gamma }(R^{-1}S^{-1}\mathrm{TR})\mathrm{dT}\;=\int_{\mathrm{SO}(T_{x}M)}(k{⋆}_{\gamma }f)(U)K_{\gamma }(R^{-1}S^{-1}U)\mathrm{dU},\;U\leftarrow \mathrm{TR}\;=((k{⋆}_{\gamma }f)⋆K_{\gamma })(\mathrm{SR}\right)$$

where we used the fact that the normalized Haar measure on $\mathrm{SO}(T_{x}M)$ is bi-invariant, thus in particular right-invariant.

Thus the following projection layer is well-defined and removes the base point and path dependency

$$f^{\ell +2}(x)=\underset{R\in \mathrm{SO}(T_{x}M)}{\max}\;F^{\left(\ell +1\right)}(x,R).$$ (7)

Biases are added per kernel. Nonlinear transformations of ReLU type are applied after each of these layers. Note that without them, a lifting followed by group correlation would actually factor in a new lifting transformation.

### 3.2 Discretization and Implementation in the Case $M=S^{2}$

In this work, the manifold of interest is $S^{2}$. Spherical functions $f:S^{2}\to R^{N}$ are typically given at a number of points and interpolated using a Watson kernel,^27^ which also serves as our choice. We use a very simple discretization of $S^{2}$ via the vertices of a regular icosahedron. Tangent kernels are defined over these vertices, sampled along with the rays of a polar coordinate system respecting the vertices of the icosahedron. The radius of the circular kernels are chosen such that when a kernel is moved from one vertex to any of its five neighbors, there is going to be overlap between the kernels before and after moving. This is illustrated in Fig. 1. We use a single-shell setup (one value at each point on the sphere) in all our experiments since it is the most common case (and it is the case for our spinal cord data). However, a multishell setup is possible if we interpolate the functions for each shell at the same locations on $S^{2}$, and the spherical function can be treated as a multichannel function.

## 4 Experiments and Results

We evaluate our method on three datasets: a DWI scan conducted on a spinal cord that had been dissected out post mortem from a deceased human female, a synthetic dataset that we generated, and the DWI brain scan dataset from the human connectome project.^28^ The human spinal cord DWI data is single-shell, with a b-value of $4000\;s/{\mathrm{mm}}^{2}$, and 80 directions per voxel. The HCP DWI data has three shells, $b=1000,2000,3000\;s/{\mathrm{mm}}^{2}$, and 90 directions per voxel. We train single-shell models, thus three separate models for the HCP data. In terms of model hyperparameter search in all experiments, we choose the hyperparameters that give us models with the lowest capacity without worsening the performance. By doing this, we get the models with proper capacity that are efficient in training meanwhile prevent overfitting. It is worth noticing that as we increase the model capacities, the stability of the models increase as well—that is, less fluctuation of loss during training, and fewer bad initiations of the models. However, we choose the least complex models possible since more complexity does not introduce better performance in this case. As for the data smoothing/interpolation parameter κ in Watson kernel, we choose the parameter values, in all experiments, that provide a trade-off between data smoothing and peak preserving. In that sense, the hyperparameters chosen are the ones that give us the best model performances.

### 4.1 Experimental Setup

After getting the responses from our proposed layers, we feed them into a small feedforward neural network—a single layer perceptron—to perform our classification task. To validate our method, we compare the proposed framework with two experimental setups: (a) a baseline experiment that feeds the smoothed signal values of each voxel directly into a feedforward neural network without our three-layer convolution; (b) S2CNN^18^ which performs convolution on spheres by transforming the signals onto the spectral domain. For all the experiments, we use the smallest model possible for both our method and S2CNN.^18^

### 4.2 Spinal Scan

#### 4.2.1 Data description

The study was conducted on a deceased individual who had bequeathed her body to science and education at the Department of Cellular and Molecular Medicine (ICMM) of the University of Copenhagen according to Danish legislation (Health Law No. 546, Section 188). The study was approved by the head of the Body Donation Program at ICMM. Part of the data used here has been published in a previous report.^29^ Briefly, the spinal cord was dissected out from a 91-year old Caucasian female without known diseases post mortem within 24 h after her death. The spinal cord was fixed by immersion into paraformaldehyde (4%), where it was kept for 2 weeks, after which it was transferred to and stored in phosphate-buffered saline until the MRI scanning was conducted. The spinal cord was placed in a plexiglas tube and immersed in fluorinert (FC-40, Sigma-Aldrich) to eliminate any background signal. The scanning was accomplished using a 9.4 T preclinical system (BioSpec 94/30; Bruker Biospin, Ettlingen, Germany) equipped with a 1.5  T/m gradient coil. The scanning was done in 29 sections of length 1.6 cm, thus covering the whole length of the spinal cord of approximately 40 cm. Between each section scan, the tissue was advanced 1.4 cm by a custom-built stepping motor system, resulting in a 0.2-cm section overlap. For each section, a T2-weighted 2D RARE structural scan was performed. Scan parameters were repetition time (TR) = 7 s, echo time (TE) = 30 ms, 20 averages, field of view $1.92\cdot 1.92\cdot 1.6\;{\mathrm{cm}}^{3}$, and a matrix size of 384·384·80, resulting in $50\cdot 50\;\mu m^{2}$ in-plane resolution and a slice thickness of 200  μm, resulting in a voxel size of $500000\;\mu m^{3}$. The scanning time for the structural scan was 30 h.

We take individual voxels containing signals defined on $S^{2}$ as the input of the networks and achieve segmentation via voxel classification. Since the numbers of samples of white matter and gray matter are not balanced, we use Focal Loss^30^ to counter the imbalance. We used 14 slices from the longest dimension to test and the rest of the scan to train.

Architecture and Hyperparameters. For our method, we use the icosahedron structure as kernel locations, and a lift-ReLU-conv-ReLU-projection-FC-softmax architecture for the network. We use k=1,5,2 channels for lift, conv, and FC, 0.6 as kernel radius, and 5 rays, 2 samples per ray as kernel resolution. For S2CNN,^18^ we use the simple architecture they provided $S^{2}\mathrm{conv}$-ReLU-SO(3)conv-ReLU-FC-softmax, bandwidth b=30,10,6 and k=4,8,2 channels. For baseline, we use FC(80)-ReLU-FC(50)-ReLU-FC(30)-ReLU-FC(2) as a multilayer perceptron alternative. We use κ=10 for the Watson kernel, 0.001 as learning rate, and trained each model for 20 epochs. We use γ=2, and α=(0.25,0.75) for white and gray matter respectively for the Focal Loss.^30^

#### 4.2.2 Results

**Fig. 2:**
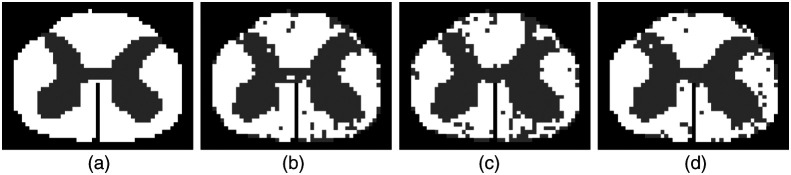
Examples of ground-truth and predictions from the test data. (a)–(d) The same slices from the ground-truth, prediction from our method, prediction from S2CNN, and prediction from baseline.

We can see from Table 1 that all methods perform quite well for this simple task. Showcase of prediction from our model and the ground-truth can be found in Fig. 2. We observe that classifying white matter and gray matter is not a challenging task considering the baseline model works well for this task. This is because there is already a significant difference between white matter and gray matter in terms of the scales of the intensity values of the two tissues. However, our method and S2CNN^18^ have a better balance between the accuracies of the two classes compared to the baseline, which shows the importance of geometric information for recognizing minority classes. To test the rotational invariance and the independence to scaling of the signals of our method, we experiment further on the synthetic dataset and the HCP dataset.^28^

**Table 1:** Results from the spinal scan. The numbers in the brackets are numbers of parameters for each model. We see that overall, all methods achieve similar performance, yet convolution involved methods—ours and S2CNN^18^—perform better in recognizing the minority class - gray matter. Numbers that are bold indicate the best result of all the methods.

Experiment (#Param)	Results		
	Our method (164)	Baseline (5802)	S2CNN (6270)
$b=4000\;s/{\mathrm{mm}}^{2}$			
Overall Acc	**0.902**	0.897	0.887
White matter Acc	0.905	**0.911**	0.891
Gray matter Acc	**0.883**	0.833	0.872

### 4.3 Synthetic Dataset

#### 4.3.1 Dataset generation

To validate the resistance of our method against rotations, we create and classify spherical functions that are defined on a sphere. We first uniformly sample 90 fixed directions on a hemisphere, and spherical functions of different classes are defined in the same 90 directions. For each class, we sample 90 values from a Gaussian distribution as function values of the 90 directions. Thus the only difference among classes is the function values of the given 90 directions, and we sample the function values for each class from the same Gaussian distribution to keep the scales of the values identical. In addition, we rotate the sphere of each class and use these rotated spherical functions as elements of each class. Therefore, each class of the dataset contains just rotations of each spherical function. As explained above, we interpolate the function values at the icosahedron vertices using a Watson kernel^27^ from the rotated 90 directions, each assigned with a function value. For the baseline, we interpolate the function values at the same 90 directions that were sampled on the sphere using the same scheme.

We generate synthetic datasets of different numbers (n∈2,4,6) of classes to test the robustness of the model, given different difficulties of the task. For each class, we generate 50 samples for the training set and 1000 samples for the test set.

*Architecture and Hyperparameters*. As in the experiment above, we use a lift-ReLU-conv-ReLU-projection-FC-softmax architecture for the network. We use k=1,5,n channels for lift, conv, and FC layers, 0.6 as kernel radius, and 5 rays, 2 samples per ray as kernel resolution. For S2CNN,^18^ we use $S^{2}\mathrm{conv}$-ReLU-SO(3)conv-ReLU-FC-softmax as in the experiments above, bandwidth b=30,10,6 and k=3,6,n channels. For baseline, we use FC(90)-ReLU-FC(50)-ReLU-FC(30)-ReLU-FC(n) as the multilayer perceptron layer structure. We use κ=5 for the Watson kernel, 0.005 as learning rate, and trained each model for 200 epochs.

#### 4.3.2 Results

See Table 2 for comparison of results from different models. We can see that the baseline model is barely learning anything from the data, while our method and S2CNN^18^ are capturing the differences from different classes in the data. Moreover, our method achieves more robust performance while having fewer degrees of freedom.

**Table 2:** Test accuracy for models evaluated on the generated datasets. Numbers in the brackets are the numbers of parameters for each model. The baseline model is producing prediction accuracies that are the same level as random guessing, while ours and S2CNN^18^ can recognize the rotations of the same spherical functions quite accurately, and our method achieves higher accuracy using fewer parameters than S2CNN.^18^ Numbers that are bold indicate the best result of all the methods.

Experiment	# Classes		
	Ours	Baseline	S2CNN
2	**1.0** (164)	0.515 (6302)	0.985 (3551)
4	**0.987** (286)	0.256 (6364)	0.966 (3565)
6	**0.984** (408)	0.168 (6426)	0.972 (3579)

### 4.4 Human Connectome Brain Scans

As in the spinal data experiments, we train networks on individual voxels containing signals on $S^{2}$. Our goal is a voxel-wise classification of four regions of the brain—cerebrospinal fluid (CSF), subcortical, white matter, and gray matter regions.

We used the preprocessed DWI data^31^ and normalized each DWI scan for the b-1000, b-2000, and b-3000 images, respectively, with the voxel-wise average of the $b_{0}$.

**Fig. 3:**
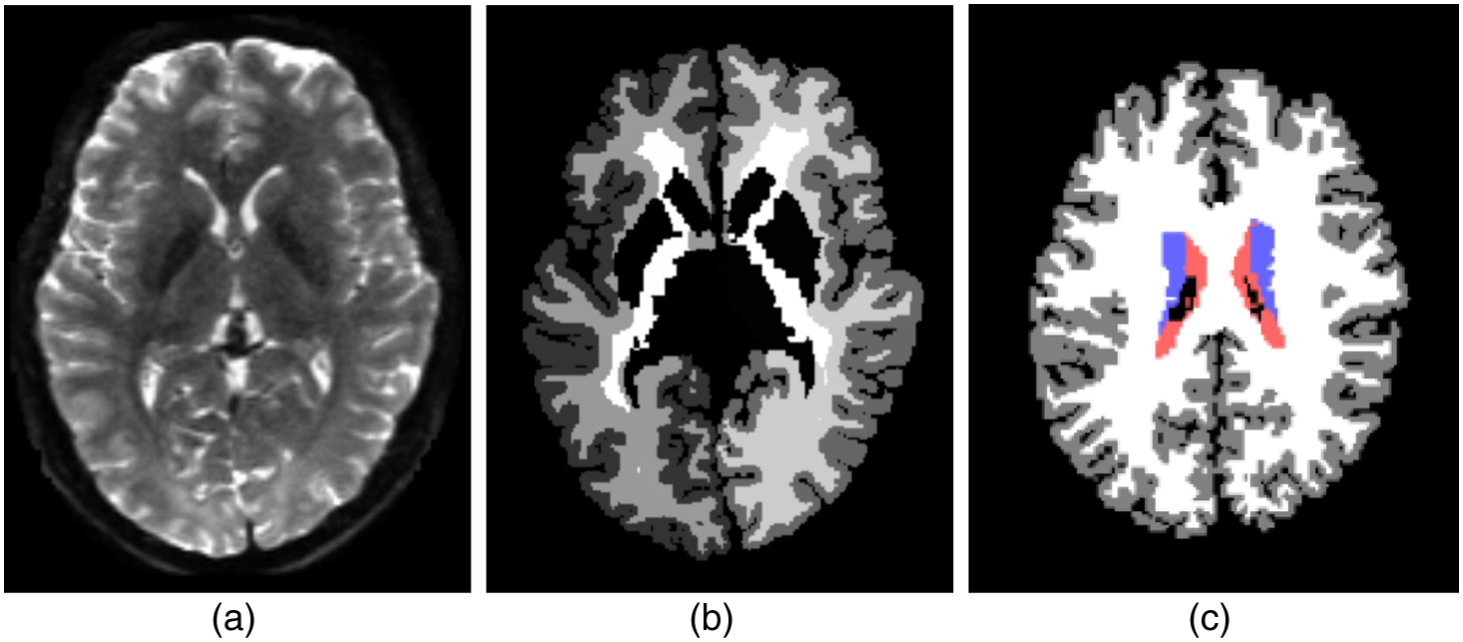
(a)–(c) original diffusion data, the ground-truth segmentation, and the processed ground-truth label image. The label colors for CSF, subcortical, white matter, and gray matter are red, blue, white, and gray, respectively. The figures are only for illustrations of the data, they are not necessarily from the same scan.

The labels provided with the T1-image were transformed to the DWI using nearest neighbor interpolation (Fig. 3). Since the four brain regions we are classifying have imbalanced numbers of voxels, we use Focal Loss^30^ to counter the class imbalance of the dataset just as in the spine data experiments.

*Architecture and hyperparameters*. As in experiments above, we use the icosahedron structure as locations for kernels for our method, and lift-ReLU-conv-ReLU-projection-FC-softmax as network architecture with k=1,5,4 channels, r=0.6 as radius, and 5 rays with 2 samples per ray as kernel resolution. For S2CNN,^18^ we again use the same architecture provided by the authors $S^{2}\mathrm{conv}$-ReLU-SO(3)conv-ReLU-FC-softmax, bandwidth b=30,10,6 and k=3,6,4 channels. For baseline, we again use FC(90)-ReLU-FC(50)-ReLU-FC(30)-ReLU-FC(4) architecture. We use κ=10 for the Watson kernel, and 0.001 as learning rate for all models. We use γ=2 and α=(0.35,0.35,0.15,0.15) for the Focal Loss^30^ for the four regions, respectively. Additionally, we have observed that the most difficult class to identify is the subcortical region, and both our method and S2CNN^18^ learn to recognize it gradually. Therefore, we stop the training for all models when the subcortical region validation accuracy stops rising. Thus we train all models for 50 epochs.

#### 4.4.1 Results

**Fig. 4:**
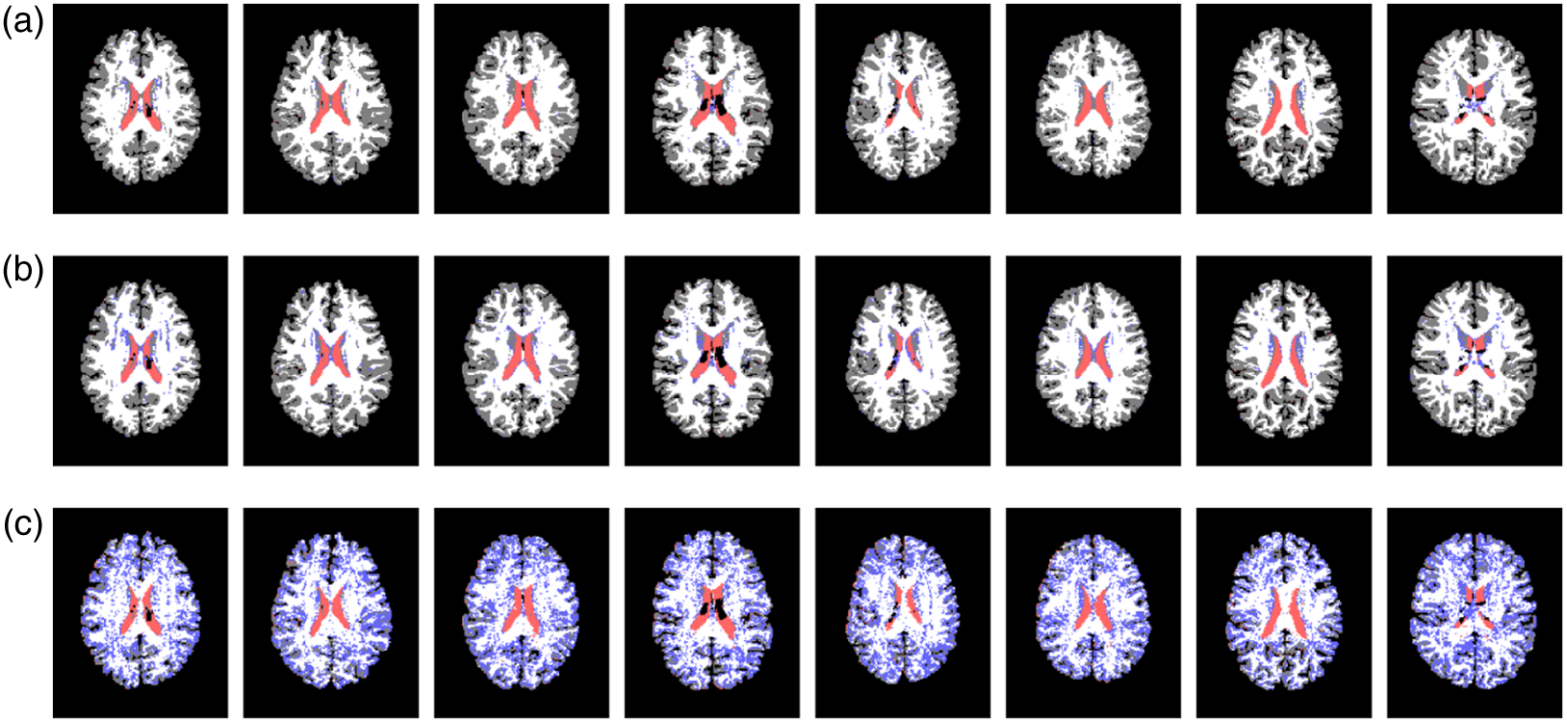
Examples of predictions of the four regions in the test set. (a)–(c) Predicted results of the same slices from the proposed method, S2CNN,^18^ and baseline respectively. The label colors for CSF, subcortical, white matter and gray matter are red, blue, white, and gray, respectively.

We used **1** scan for training, **1** scan for validation, and **50** scans for testing. We chose this split of the dataset for training/validation/testing because it is the most light-weight for training. Including more scans in the training set was also tried, but it did not seem to make the results much better. Therefore, we only used one scan in the training set in the end. Comparison of experimental results of different methods can be found in Table 3. We can see that the baseline experiment does not generalize well compared to our method and S2CNN.^18^ Across different b values, we observe that with increased b, over all experiments, it becomes harder to recognize CSF. Higher b does not reduce the accuracies for the majority classes for our method and S2CNN,^18^ thus the overall accuracies from these methods do not drop much with increased b. On the other hand, while comparing to S2CNN,^18^ we achieve very similar results yet our model has way lower degrees of freedom while achieving the same level of performance as we can see in Table 3. Showcases of predictions from all models can be found in Fig. 4.

**Table 3:** Results from the HCP brain dataset. We can see that our method has the same level of performance as S2CNN,^18^ but uses way fewer parameters. The baseline model produces higher accuracy recognizing the subcortical region, but it is at a high cost of the accuracies of other classes.

Experiment (#Param)	Results		
	Our method (286)	Baseline (6364)	S2CNN (3565)
$b=1000\;s/{\mathrm{mm}}^{2}$			
Overall Acc	0.791±0.012	0.492±0.015	0.784±0.012
CSF Acc, Dice	0.785±0.074, 0.747±0.073	0.824±0.06, 0.577±0.106	0.783±0.075, 0.744±0.074
Subcortical Acc, Dice	0.201±0.057, 0.239±0.052	0.495±0.031, 0.128±0.011	0.299±0.059, 0.276±0.039
White matter Acc, Dice	0.778±0.026, 0.802±0.014	0.67±0.016, 0.691±0.012	0.816±0.023, 0.81±0.012
Gray matter Acc, Dice	0.872±0.017, 0.835±0.011	0.327±0.026, 0.483±0.028	0.814±0.02, 0.827±0.012
$b=2000\;s/{\mathrm{mm}}^{2}$			
Overall Acc	0.787±0.012	0.452±0.017	0.794±0.011
CSF Acc, Dice	0.552±0.075, 0.612±0.078	0.76±0.07, 0.605±0.098	0.753±0.079, 0.684±0.088
Subcortical Acc, Dice	0.184±0.045, 0.222±0.042	0.694±0.021, 0.144±0.008	0.123±0.03, 0.166±0.034
White matter Acc, Dice	0.804±0.032, 0.806±0.015	0.689±0.022, 0.748±0.015	0.843±0.025, 0.817±0.012
Gray matter Acc, Dice	0.85±0.023, 0.827±0.012	0.207±0.027, 0.339±0.036	0.832±0.021, 0.83±0.011
$b=3000\;s/{\mathrm{mm}}^{2}$			
Overall Acc	0.786±0.012	0.686±0.016	0.788±0.011
CSF Acc, Dice	0.203±0.029, 0.284±0.04	0.188±0.014, 0.222±0.028	0.303±0.055, 0.341±0.069
Subcortical Acc, Dice	0.216±0.057, 0.248±0.05	0.358±0.023, 0.186±0.015	0.228±0.064, 0.256±0.055
White matter Acc, Dice	0.767±0.034, 0.805±0.018	0.83±0.021, 0.797±0.011	0.783±0.033, 0.812±0.016
Gray matter Acc, Dice	0.888±0.02, 0.832±0.011	0.616±0.037, 0.714±0.024	0.873±0.021, 0.831±0.012

**Table 4:** Results of sensitivity analysis. The numbers in the first row are the numbers of samples in each experiment for CSF, subcortical, WM, and GM, respectively. We see that while reducing the size of the training set, the overall accuracies decrease to some extent, but the accuracies of the subcortical region are higher since the class imbalance is lower.

#Samples	Results		
	5000, 30,000, 200,000, 200,000	5000, 30,000, 150,000, 150,000	5000, 30,000, 75,000, 75,000
$b=1000\;s/{\mathrm{mm}}^{2}$			
Overall accuracy	0.784±0.013	0.775±0.014	0.718±0.024
CSF accuracy, Dice	0.719±0.086, 0.747±0.073	0.776±0.076, 0.752±0.073	0.853±0.055, 0.704±0.082
Subcortical accuracy, Dice	0.271±0.072, 0.279±0.051	0.289±0.056, 0.247±0.032	0.643±0.061, 0.293±0.025
White matter accuracy, Dice	0.736±0.026, 0.791±0.015	0.746±0.029, 0.789±0.016	0.635±0.042, 0.733±0.026
Gray matter accuracy, Dice	0.887±0.017, 0.832±0.012	0.857±0.018, 0.834±0.012	0.794±0.028, 0.819±0.017
$b=2000\;s/{\mathrm{mm}}^{2}$			
Overall accuracy	0.777±0.014	0.771±0.017	0.729±0.017
CSF accuracy, Dice	0.711±0.089, 0.695±0.087	0.744±0.079, 0.682±0.088	0.772±0.074, 0.676±0.09
Subcortical accuracy, Dice	0.24±0.039, 0.235±0.028	0.362±0.061, 0.293±0.033	0.462±0.043, 0.24±0.018
White matter accuracy, Dice	0.737±0.033, 0.789±0.018	0.735±0.037, 0.789±0.02	0.716±0.031, 0.78±0.018
Gray matter accuracy, Dice	0.876±0.019, 0.83±0.012	0.851±0.022, 0.826±0.013	0.769±0.031, 0.802±0.015
$b=3000\;s/{\mathrm{mm}}^{2}$			
Overall accuracy	0.785±0.011	0.772±0.013	0.662±0.023
CSF accuracy, Dice	0.603±0.106, 0.584±0.112	0.525±0.082, 0.528±0.102	0.701±0.09, 0.568±0.112
Subcortical accuracy, Dice	0.288±0.068, 0.277±0.047	0.388±0.068, 0.295±0.037	0.63±0.048, 0.222±0.018
White matter accuracy, Dice	0.798±0.028, 0.809±0.013	0.761±0.028, 0.801±0.014	0.596±0.038, 0.719±0.027
Gray matter accuracy, Dice	0.836±0.026, 0.829±0.012	0.833±0.027, 0.826±0.012	0.722±0.042, 0.78±0.02

**Model Sensitivity Analysis**. We reduce the amount of training data for our method in order to test how sensitive our model is. As mentioned above, there is only one scan in the training set. For that scan, there are 7227 CSF voxels, 35,648 subcortical voxels, 276,191 white matter voxels, and 309,496 gray matter voxels. Therefore, we reduce the number of samples in the training scan from all classes by randomly sampling a fraction of voxels from each class and test how that impacts the performance of the model. The results can be found in Table 4.

We see that reducing the number of samples in each class reduces the performance. On the other hand, it has also boosted the accuracy for the subcortical region, since that the class imbalance was also eased after the reduction. We can observe that the gray matter and white matter tissues are overly represented in a scan that even when we discard most of the voxels from these two classes in the training set, our test result remains a relatively high level of accuracy. This offers us an important application in automating DWI data annotation.

## 5 Discussion

This work shows how geometric information in DWI can be significantly useful in understanding general patterns in image analysis. In the future, we expect improvements in performance by adding spatial correlations—convolutions in the product space $R^{3}\times S^{2}$ instead of mere $S^{2}$, for example. This is ongoing work. The correlation of our model to fractional anisotropy (FA) and NODDI is worth investigating as well. Moreover, using scans in the HCP dataset^28^ with a different number of diffusion gradients to test our model would also be desirable in later works. Our model generalizes well to the test set while trained with very little data (one scan), but this generalization is limited to data with the same distribution, i.e., with acquisitions from the same scanner using the same protocol. A new dataset with a new acquisition protocol would require new training. It will be desirable to apply our model to datasets that consist of irregular scans such as brains with tumors and unprocessed scans, unlike the HCP dataset which only has preprocessed healthy brains. Obtaining that kind of data is another challenge. Additionally, we have so far only tested our construction of the network on $S^{2}$, yet an extension to other surfaces appears feasible, with a smart choice of a discrete representation. An extension to dimension 3 is worthwhile as well, which will require efficient SO(3) convolutions, using, for instance, spectral theory for compact Lie groups.

## 6 Conclusion

We proposed a simple extension of CNN to Riemannian Manifolds that learns rotationally invariant features. Our method allows us to learn general patterns from very limited data while having much lower degrees of freedom than existing methods.^18^ This is significant because we can now, in machine learning-based DWI analysis, reduce the size of individual data samples to a single voxel-level from a whole volumetric image-level, as well as reduce the training dataset to a single scan—or a fraction of a scan. For the HCP dataset^28^ with a single-shell setup, our method, while taking the subcortical region into account, compares well with existing methods that have multishell input,^32^^,^^33^ which do not classify the subcortical region. We also achieved similar or better results compared to image registration-based methods.^34^ The results of this simple task show great potential of this method in understanding structural patterns in brains. Moreover, the results from the model sensitivity analysis show that our method has the potential in aiding manual data annotation. For example, a doctor only has to label a fraction of a scan and the rest can be automated by the model.
